# A Rare Case Report of an Adult With Intraspinal Gouty Stones and Concurrent Knee Joint Involvement

**DOI:** 10.1002/ccr3.70573

**Published:** 2025-07-02

**Authors:** Bo Cao, Jinlong Wang

**Affiliations:** ^1^ Department of Orthopedics Center Affiliated Qingyuan Hospital, The Sixth Clinical Medical School, Guangzhou Medical University, Qingyuan People's Hospital Qingyuan China; ^2^ Department of Neurosurgery Binzhou Medical University Affiliated Shengli Oilfield Central Hospital Dongying China

**Keywords:** gouty arthritis, knee gout, knee joint, lumbar fusion, spinal gout

## Abstract

This case highlights the importance of considering spinal gout in patients with hyperuricemia presenting with atypical back pain or neurological symptoms. Multidisciplinary surgical management, including decompression and joint arthroscopy, combined with urate‐lowering therapy and lifestyle modifications, can achieve significant symptom relief and prevent disease recurrence.

## 1 Introduction

Gout is a chronic, systemic disease caused by monosodium urate (MSU) crystal deposition in tissues and affects mainly the joints [1]. In contrast, spinal gout, the deposition of MSU crystals in the vertebral body, is very rare, accounting for less than 1% of cases in some studies [2, 3]. The formation of intraspinal tophi, cast‐like deposits in the spinal canal or vertebral body that can mimic other spinal pathologies, such as tumors, infections, and degenerative diseases, in gout is even rarer [4, 5].

This case report describes a very unusual manifestation of gout. The patient presented with tophi in the spinal canal, and gout in the knee. Although spinal gout is rare, tophi that actually form in the vertebral body are rarer, increasing the complexity of diagnosis and treatment. In this patient, spinal involvement was the initial leading cause due to severe back pain and radicular symptoms, after lumbar fusion and decompressive surgery, the patient experienced a knee arthroscopy for gout stone removal, highlighting the systemic nature of the disease.

The challenges in this case were multifaceted, including the diagnostic difficulty in differentiating spinal gout and the therapeutic challenge in dealing with severe postoperative recurrence in distant joints. This case highlights the need to be highly vigilant for the manifestations of atypical gout, especially in patients with a history of hyperuricemia, and the importance of careful postoperative management to prevent the recurrence of gout.

## 2 Case History/Examination

The patient was a 22‐year‐old man who complained of persistent back pain, numbness and weakness in his right lower extremity for 6 months. His symptoms gradually worsened, eventually leading to difficulty walking and performing activities of daily living. The patient reported a history of gout and two episodes of heel pain in the past two years but did not receive systemic treatment for the conditions. The initial uric acid level was substantially elevated (836 μmol/L). At the time of medical treatment, he was not receiving any uric acid‐lowering treatment, and his diet was full of purine‐rich foods. Notably, there was no evidence of extraspinal tophi on clinical or imaging evaluations. Additionally, there was no reported family history of gout or hyperuricemia, and no known hereditary causes of hyperuricemia were identified.

**FIGURE 1:**
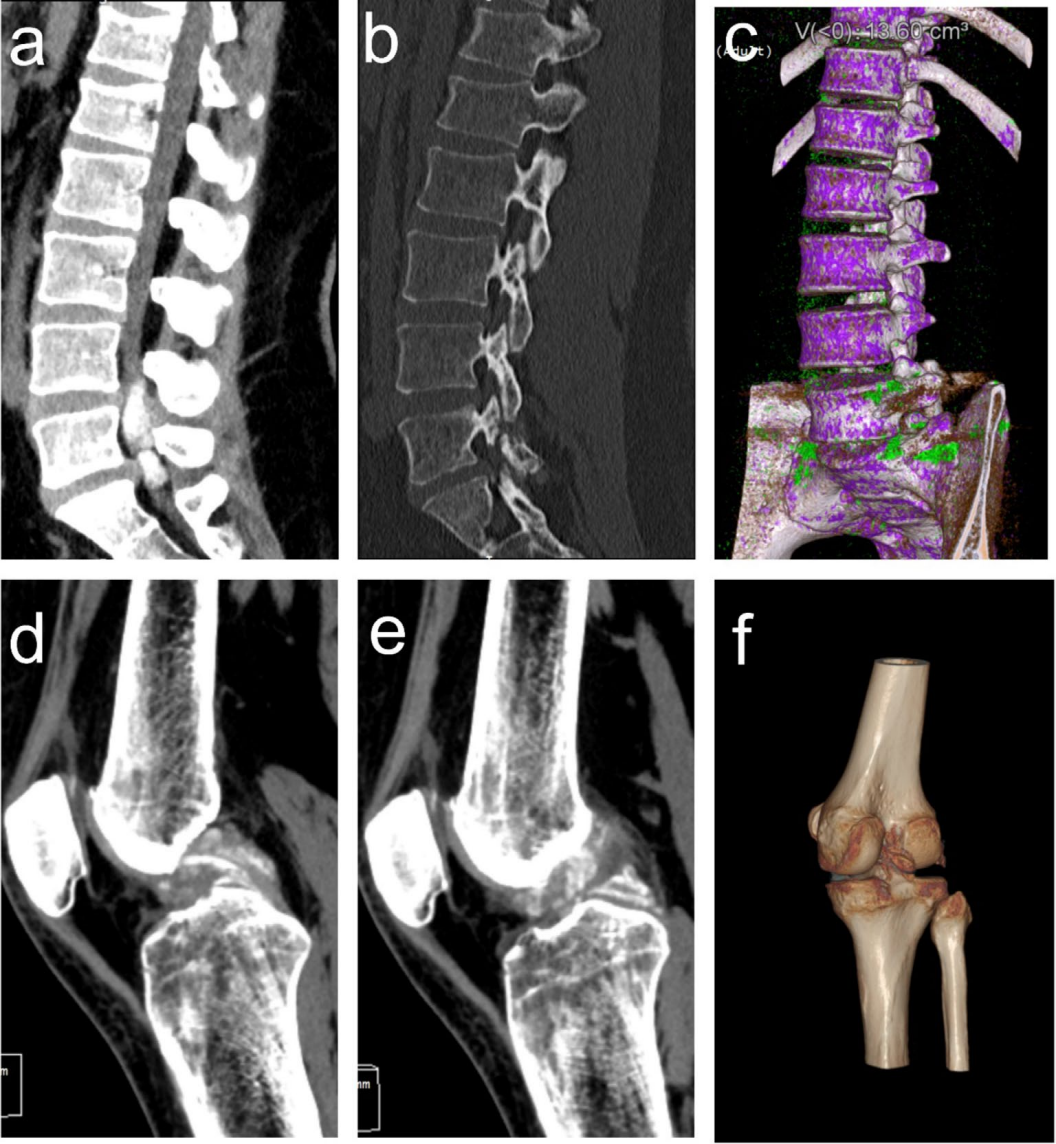
CT Scan of the Spine and knee. (a) CT image showing the presence of gouty granulomas within the spinal canal at the L5‐S1 level. (b) CT scan showing the isthmic fracture and the disc herniation at the L5‐S1 level. Notable findings include changes in bone density and the presence of gouty stones. (c) Dual‐Energy CT (DECT) showing the presence of gouty stones. (d–f) (DECT) a large number of gouty stones in the knee joints.

**FIGURE 2:**
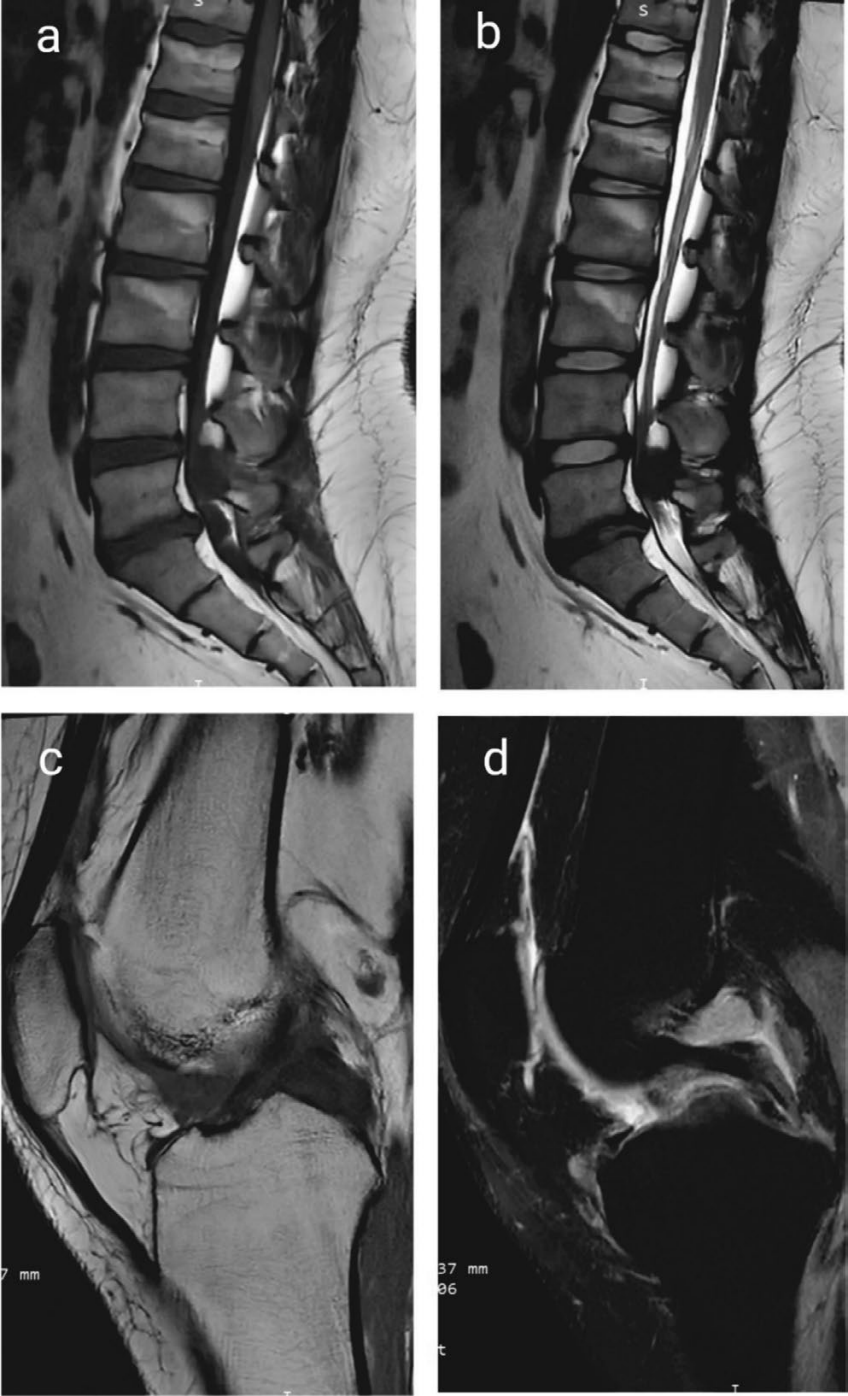
MRI of the spine and knee. (a) CT image showing the presence of gouty granulomas within the spinal canal at the L5‐S1 level. (b) CT scan showing the isthmic fracture and the disc herniation at the L5‐S1 level. Notable findings include changes in bone density and the presence of gouty stones. (c) Sagittal MRI of the left knee showing intra‐articular tophaceous deposits around the femoral condyle and joint space. (d) MRI of the knee demonstrating extensive gouty tophi in the anterior and posterior cruciate ligaments.

During the physical examination, the patient experienced obvious numbness and a tingling sensation in the right lower extremity, especially in the outer side of the calf and the large toe, and the range of motion in the right knee was limited. Neurological examination revealed that the patient had mild muscle weakness and hypoesthesia at the L5‐S1 dermatomes. CT (Figure 1) and MR (Figure 2) of the right knee showed extensive gouty stones in the anterior–posterior crossing of the right knee. Musculoskeletal examination revealed tenderness in the right iliacus muscle, and the right knee was slightly bent due to pain, with mild limitation of right knee flexion and extension on right knee examination.

Magnetic resonance imaging (MRI) of the lumbar spine revealed a mass in the spinal canal at L4‐L5 (Figure 2a,b), nerve root compression, and calcified deposits in the lesion, which involved the facial joints and was suspected to be within the spinal canal. Non‐contrast enhanced computed tomography (CT) (Figure 1) confirmed the existence of multiple high‐density lesions, suggesting the presence of tophi in the spinal canal. Dual‐energy CT (DECT) (Figure 1c) of the postoperative spine and preoperative knee intraoperative pathology further confirmed gouty stones in the joints and spine. Laboratory examination (Table 1) revealed increased serum uric acid levels (836 μmol/L) and mildly elevated liver enzymes (alanine aminotransferase (ALT): 243 U/L, aspartate aminotransferase (AST): 113 U/L).

**TABLE 1:** Identified gene mutations.

Gene	Chromosome position
Age	22 years
Gender	Male
Occupation	Office worker
Medical history Initial symptoms	History of gout Back pain, lower extremity weakness
Uric acid level	836 μmol/L

## 3 Differential Diagnosis

DECT (Figure 1) is crucial for identifying MSU crystal deposition. MRI and CT distinguish between mechanical, infectious, and neoplastic causes of spinal lesions. Laboratory tests: Elevated serum uric acid strongly supports gout, while markers like CRP and ESR help rule out infections or systemic inflammation. Definitive diagnosis in this case was made through surgical tissue analysis, confirming MSU crystals.

## 4 Outcome and Follow‐Up

The patient underwent surgical decompression and resection of the spinal canal lesion via laminectomy. The procedure lasted approximately 6 h, with meticulous dissection required due to the extensive involvement of the L5 nerve root. During surgery, white chalk‐like deposits were observed in the spinal canal, which were consistent with MSU crystals (Video 1). The MSU crystals were densely adherent to the dura and surrounding neural structures. Video 1 demonstrates surgical decompression and resection of the spinal canal lesion via laminectomy.

**VIDEO 1:** The intraoperative visualization of gout stones region is clearly visible. Intraoperative exploration revealed bilateral lumbar 5 and right sacral 1 spinal cord nerve roots encapsulated by gouty granulomatous tissue adhesions. Video content can be viewed at https://onlinelibrary.wiley.com/doi/10.1002/ccr3.70573

The L5 nerve root was wrapped by gout stones, causing severe compression. Surgery included removal of these deposits and an L5‐S1 disc herniation. One of the primary surgical challenges was the dense adherence of the gouty deposits to the dura, raising concerns about potential dural tears or cerebrospinal fluid (CSF) leakage. Careful microdissection techniques, including the use of microsurgical instruments and saline irrigation, were employed to progressively separate the deposits from the dura while minimizing traction on the nerve root. Additionally, the L5 nerve root was completely encased by the tophaceous material, causing severe compression. A combination of microsurgical debulking, ultrasonic aspiration, and blunt dissection was utilized to decompress the nerve while preserving its integrity.

Another significant challenge was the coexistence of an L5‐S1 disc herniation, which compounded the nerve compression. After the resection of the tophi, a standard microdiscectomy was performed to relieve additional pressure on the nerve root. Throughout the procedure, particular attention was paid to hemostasis due to the friable nature of the surrounding tissue, reducing the risk of postoperative epidural hematoma.

Due to the chronic inflammation and fibrosis associated with spinal gout, there was an increased risk of dural injury and nerve manipulation‐related deficits. In this case, while some segments of the dura were partially adherent to the tophaceous deposits, meticulous dissection with sharp and blunt techniques allowed for successful separation without CSF leakage. The spinal cord and nerve roots were continuously monitored for signs of excessive traction or ischemic injury.

Postoperatively, the patient underwent neurological monitoring and rehabilitation, with an emphasis on assessing motor and sensory function in the L5‐S1 distribution. The risk of recurrence remains a concern, emphasizing the importance of long‐term urate‐lowering therapy to prevent further MSU deposition.

**FIGURE 3:**
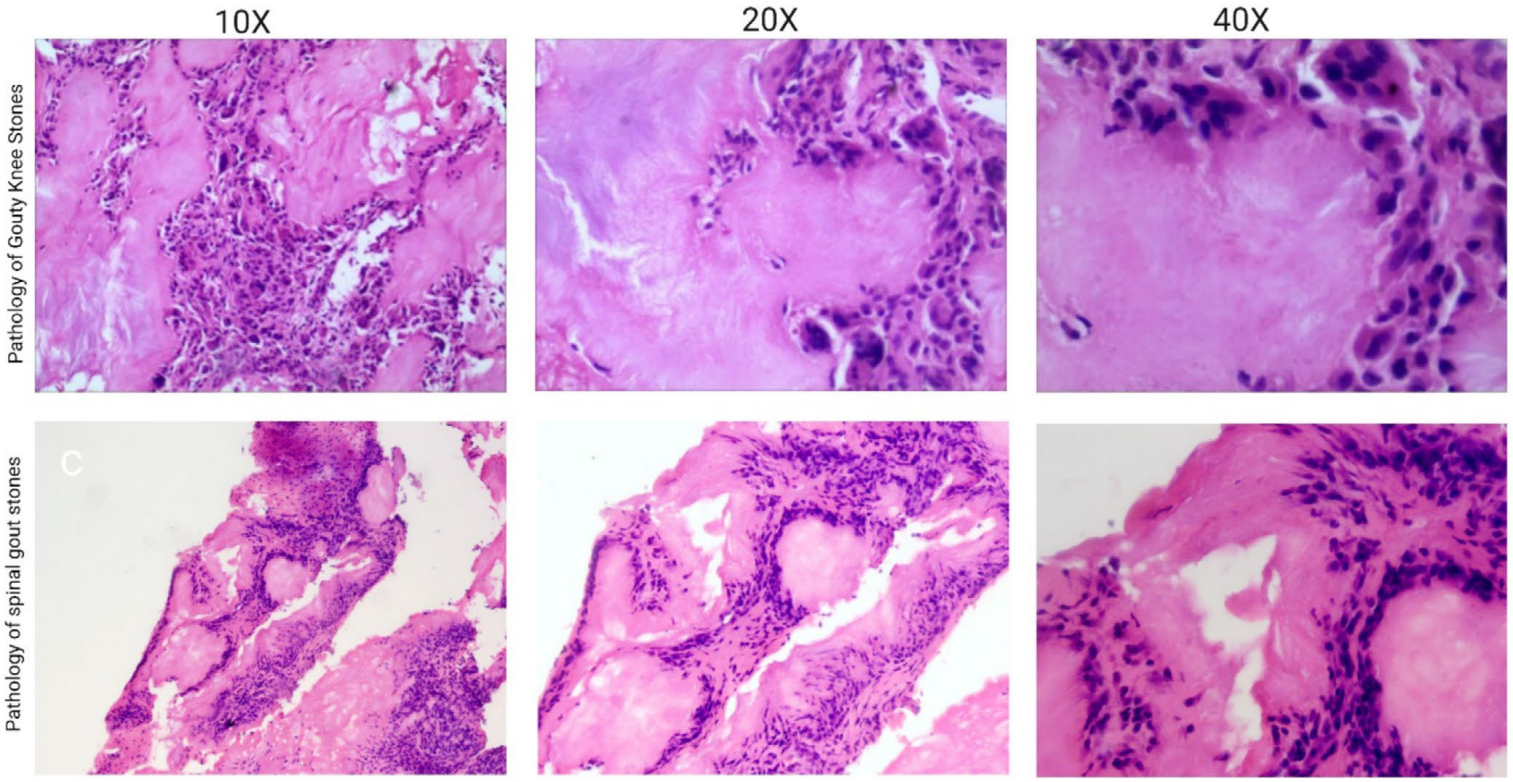
Intraoperative histopathological images of gout. Intraoperative gouty histopathological images showed structures consistent with gouty foreign body granuloma.

Pathological analysis of the resected tissue (Figure 3) confirmed the presence of MSU crystal deposition, surrounded by chronic inflammatory changes and fibrosis, consistent with advanced tophaceous gout.

**FIGURE 4:**
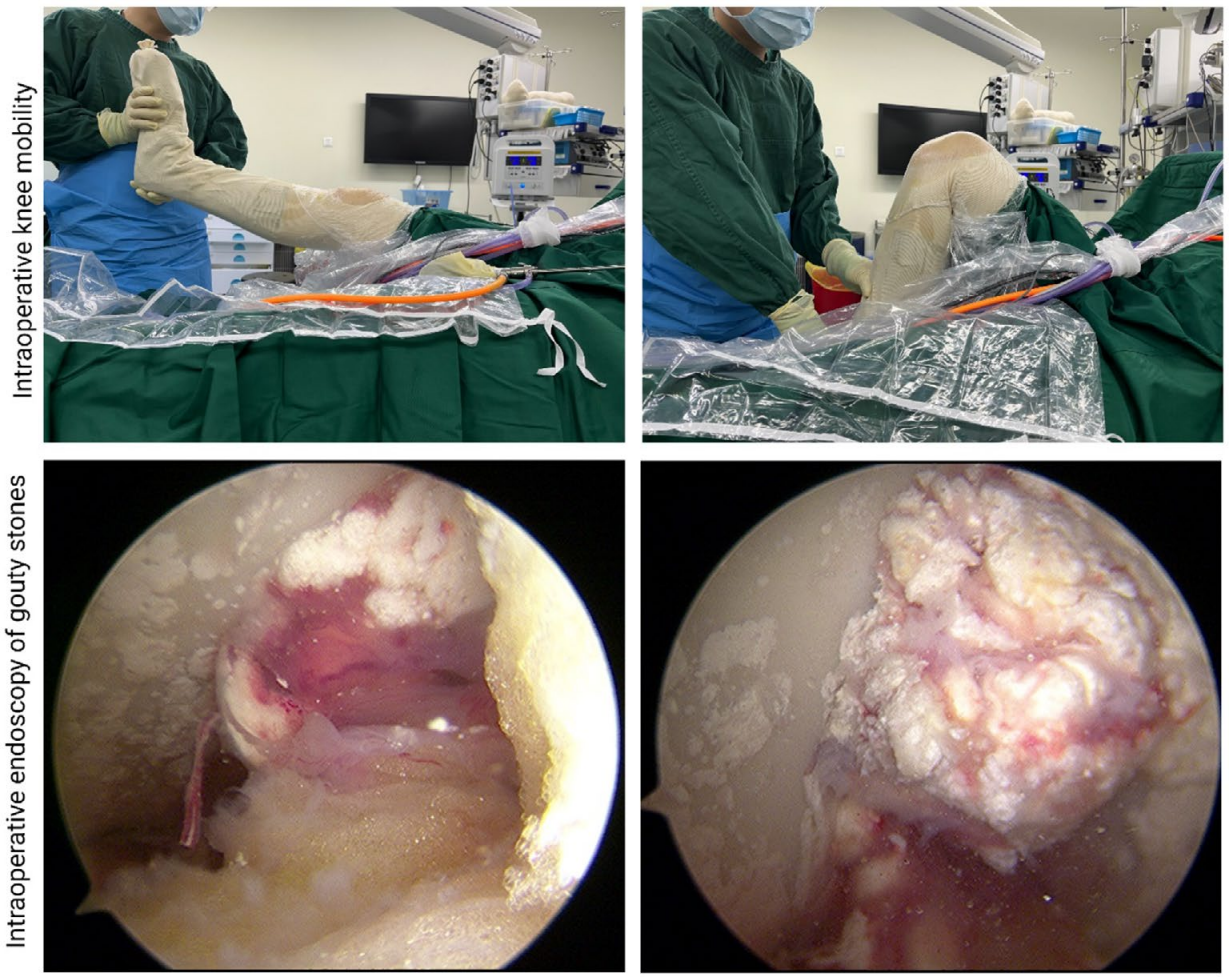
Intraoperative images from the surgical procedure. Intraoperative detection of knee flexion and extension. Intraoperative endoscopy revealed a large number of gouty stones around the anterior and posterior cruciate ligaments of the knee.

The patient's right knee underwent knee arthroscopy (Figure 4) for gouty stone removal. A large number of gouty stones were seen around the anterior and posterior cruciate ligaments on intraoperative endoscopy. Intraoperative pathology further confirmed gouty stones.

After surgery, the patient received comprehensive treatments (Table 2) aimed at controlling inflammation, managing uric acid levels, and promoting functional recovery. A nonsteroidal anti‐inflammatory drug (NSAID), loxoprofen sodium, was used to alleviate inflammation and pain. Uric acid‐lowering treatment: The patient started to take febuxostat 20 mg per day, and the dose was gradually increased according to the subsequently measured serum uric acid level. To prevent postoperative stiffness and enhance mobility, the patient was encouraged to perform active range‐of‐motion exercises of the spine and knee joints to prevent stiffness and improve mobility. In addition, the patient was instructed to perform low‐intensity aerobic exercise to maintain joint flexibility and avoid aggravating symptoms.

**TABLE 2:** Postoperative management strategies.

Strategy	Details
NSAIDs	Indomethacin 50 mg daily for pain management
Corticosteroids	Methylprednisolone intra‐articular injection in knee
Urate‐lowering therapy	Allopurinol, dosage adjusted based on uric acid levels
Physical rehabilitation	Range of motion exercises, strengthening

During hospitalization, the patient's back pain and numbness in the lower extremities were significantly alleviated, and his knee symptoms gradually resolved after intra‐articular steroid injection. When discharged from the hospital, the doctors advised him to continue taking NSAIDs and febuxostat and recommended that he regularly monitor his liver function and serum uric acid levels to ensure long‐term safety and efficacy. At the three‐month follow‐up, the patient exhibited no signs of recurrent intraspinal, reported sustained pain relief, and demonstrated improved spinal and joint mobility. Serum uric acid levels remained within the target range, and medication adherence was consistent without reported adverse effects. Given the chronic and recurrent nature of gout, extended follow‐up was recommended to monitor for potential disease recurrence, assess neurological function, and ensure continued adherence to pharmacological and lifestyle interventions.

## 5 Discussion

Spinal gout is a rare condition, and its diagnosis is highly challenging because of its nonspecific symptoms and its clinical and radiological resemblance to other spinal pathologies, such as infectious spondylodiscitis, neoplastic lesions, or degenerative changes [6]. A literature review revealed that the incidence of spinal and knee gout is less than 1% among gout patients [7]. Therefore, spinal gout should be considered as uncommon for patients presenting but an important differential diagnosis for patients with back pain or spinal cord compression. S. A. Wan et al. reported that the incidence of spinal canal involvement in gout is even lower than previously estimated [8]. These tophi (or vertebral stones) may form in different locations of the spine, but especially in the lumbar spine, as in this case [9]. Although there are sporadic reports of gout stones affecting the spine, this case is unique since the stones were in the spinal canal, which poses a challenge to the diagnosis [10]. This condition is usually associated with more common spine diseases, such as tumors or infections. Few studies have investigated the exact incidence of this type of spinal lesion; therefore, this case is a rare and valuable addition to the medical literature on gout‐associated spine diseases [11].

Given its rarity, spinal gout can easily be misdiagnosed as other more common spinal disorders. Infectious spondylodiscitis typically presents with fever, elevated inflammatory markers, and vertebral endplate destruction on MRI, whereas spinal gout lacks systemic signs of infection and often exhibits well‐demarcated, calcified deposits on CT [8]. Neoplastic lesions, such as spinal metastases or primary bone tumors, may mimic spinal gout radiologically but are more likely to demonstrate aggressive bone destruction, contrast enhancement, and soft tissue mass formation on MRI [9]. Degenerative changes, including spondylosis or herniated discs, often cause nerve compression, but these conditions typically show disc dehydration, osteophyte formation, and facet joint hypertrophy rather than focal calcified masses. The presence of hyperuricemia, a history of gout, and DECT findings can help differentiate spinal gout from these other conditions.

Interestingly, the patient experienced a concurrence of gout in the knee joint, indicating that there was a systemic reaction during surgical resection of the tophi in the spinal canal. Systemic inflammation may mobilize the original MSU deposits in the peripheral joints, resulting in an acute attack of gout. This “migrating seizure” phenomenon is a well‐recognized but poorly understood complication, especially during systemic inflammation [12].

Gouty stone formation within the spine, as opposed to peripheral joints, raises intriguing pathophysiological questions. While hyperuricemia is the primary factor in MSU crystal deposition, the specific predilection for the L5‐S1 level in this case warrants further exploration. One hypothesis is that the biomechanical stress and relatively lower vascularity of the lower lumbar spine contribute to local microenvironmental changes that favor MSU deposition. However, this remains speculative, and additional studies are needed to delineate the precise mechanisms underlying spinal tophi formation.

The clinical manifestations of spinal gout are nonspecific and may be similar to those of other conditions, such as spinal infection, tumors, and degenerative diseases; therefore, it is very difficult to differentially diagnose spinal gout [13]. In this case, the initial symptoms of back pain and numbness of the lower extremities led to an extensive differential diagnosis, including a spinal tumor or disc herniation, based on the findings of MRI and CT scanning. However, imaging examinations also revealed calcified lesions in the vertebral body, which, together with the patient's hyperuricemia and gout history, prompted the doctors to further investigate the cause of gout mimicking chondrosarcoma.

Radiological imaging plays a critical role in managing gout, but conventional imaging modalities is often not sufficient for diagnosis [14]. Spinal lesions caused by gout may not present with the typical imaging features of gout in the peripheral joints, such as perforating erosions and overhanging edges. In this case, the high‐density calcifications observed on CT imaging helped to differentiate gout from other spinal lesions, and intraoperative findings and histopathological analysis confirmed this diagnosis. And DECT has emerged as a promising tool for detecting MSU crystal deposits, but its diagnostic accuracy in spinal gout remains underexplored. A systematic comparison of DECT with conventional CT and MRI in differentiating spinal gout from other spinal lesions would be beneficial for future clinical practice.

This case highlights the challenges in diagnosing and treating spinal gout, especially the rare occurrence of MSU crystals in the spinal canal. However, this study describes only one patient, limiting the generalizability of the findings. The rarity of concurrent intraspinal tophi and peripheral joint flares makes it challenging to draw broad clinical conclusions or to establish causative factors confidently. We present limited longitudinal data on post‐treatment outcomes and management efficacy over time. More extended follow‐up with this patient would be valuable to evaluate the effectiveness and recurrence risk associated with the selected interventions, including pharmacological and surgical treatments.

Future research on spinal gouty stones with concurrent peripheral joint involvement, such as knee tophi, could focus on several key areas. Multicenter case series, compiling patient characteristics, spinal involvement patterns, treatment modalities, and outcomes, would be instrumental in gathering a comprehensive dataset, aiding in the establishment of diagnostic and treatment standards. Furthermore, studies exploring the pathophysiology of MSU crystal deposition in the spine versus peripheral joints could offer mechanistic insights into why specific spinal levels, such as L5‐S1, are preferentially affected.

To enhance the clinical relevance of this case report, we have included a summary table (Table 3) comparing previously reported cases of spinal gout, detailing patient characteristics, spinal involvement, and treatment outcomes. This addition strengthens the discussion by contextualizing our case within the broader literature and providing clinicians with a comparative perspective on spinal gout presentations.

**TABLE 3:** Comparing previously reported cases of spinal gout.

Study/Case report	Age/Sex	Spinal level involvement	Symptoms	Diagnostic method	Treatment	Outcome
Current Case	45/M	L5‐S1	Back pain, radiculopathy, concurrent knee gout	CT, MRI, DECT, histopathology	Surgical decompression, febuxostat, NSAIDs	Symptom resolution, no recurrence at short‐term follow‐up
Wan et al. (2021) [15]	52/M	L3‐L4	Lower limb weakness, numbness	MRI, biopsy	Laminectomy, urate‐lowering therapy	Partial symptom resolution
Kim et al. (2018) [16]	60/F	L4‐L5	Severe back pain, sciatica	CT, MRI	Laminectomy, tophi resection	Full recovery
Chen et al. (2015) [17]	48/M	C5‐C6	Quadriparesis	CT, DECT	Surgical decompression	Significant improvement
Pettit et al. (2013) [18]	55/M	T12‐L1	Progressive myelopathy	MRI, biopsy	Conservative (urate‐lowering therapy, NSAIDs)	Symptom stabilization
Zhang et al. (2010) [19]	67/M	L2‐L3	Paraplegia	CT, biopsy	Laminectomy, colchicine	Limited recovery

This report adds valuable insights to the growing recognition of atypical gout presentations, stressing the need for early detection, accurate diagnosis, and comprehensive care strategies for individuals with multi‐joint involvement. It also suggests potential areas for future research to understand the pathophysiology, optimal management, and long‐term outcomes of gout.

## 6 Conclusions

This case of intraspinal tophi with concurrent knee joint gout highlights key clinical considerations for diagnosing and managing rare manifestations of gout. While spinal gout is infrequently encountered, it should be considered in patients with hyperuricemia and atypical back pain or neurological symptoms. Misidentification of MSU deposits in the spinal canal is possible, as these can mimic common spinal lesions. Therefore, a combination of advanced imaging modalities—notably MRI and CT—paired with a detailed medical history and laboratory evaluation is recommended to enhance diagnostic accuracy.

## Author Contributions

**Bo Cao:** project administration, supervision, writing – review and editing. **Jinlong Wang:** data curation.

## Ethics Statement

Ethics approval for this study was obtained from the Ethics Board of Shengli Oilfield Central Hospital. The ethics code: YXLL202412501.

## Consent

Written informed consent was obtained from the patient for publication of this case report and any accompanying images. A copy of the written consent is available for review by the Editor‐in‐Chief of this journal.

## Conflicts of Interest

The authors declare no conflicts of interest.

## Data Availability

The data that support the findings of this study are available from the corresponding author upon reasonable request.
